# Retention of normal glia function by an isoform-selective protein kinase inhibitor drug candidate that modulates cytokine production and cognitive outcomes

**DOI:** 10.1186/s12974-017-0845-2

**Published:** 2017-04-05

**Authors:** Zhengqiu Zhou, Adam D. Bachstetter, Claudia B. Späni, Saktimayee M. Roy, D. Martin Watterson, Linda J. Van Eldik

**Affiliations:** 1 Sanders-Brown Center on Aging, University of Kentucky, 800 S. Limestone Street, Lexington, KY USA; 2 Spinal Cord and Brain Injury Research Center, University of Kentucky, 741 S. Limestone Street, Lexington, KY USA; 3 Department of Neuroscience, University of Kentucky, 800 Rose Street, Lexington, KY USA; 4 Department of Pharmacology, Northwestern University, 303 E Chicago Ave, Chicago, IL USA

**Keywords:** Cytokines, Microglia, Protein kinase inhibitors, Mitogen-activated protein kinase 14, Neuroprotective agents, Alzheimer’s disease

## Abstract

**Background:**

Brain p38α mitogen-activated protein kinase (MAPK), a potential therapeutic target for cognitive dysfunction based on the neuroinflammation-synaptic dysfunction cycle of pathophysiology progression, offers an innovative pharmacological strategy via inhibiting the same activated target in both glia and neurons, thereby enhancing the possibility for efficacy. The highly selective, brain-penetrant p38αMAPK inhibitor MW150 attenuates cognitive dysfunction in two distinct Alzheimer’s disease (AD)-relevant models and avoids the problems encountered with previous mixed-kinase inhibitor drug candidates. Therefore, it is essential that the glial effects of this CNS-active kinase inhibitor be addressed in order to anticipate future use in clinical investigations.

**Methods:**

We explored the effects of MW150 on glial biology in the AD-relevant APP/PS1 knock-in (KI) mouse model where we previously showed efficacy in suppression of hippocampal-dependent associative and spatial memory deficits. MW150 (2.5 mg/kg/day) was administered daily to 11–12-month-old KI mice for 14 days, and levels of proinflammatory cytokines IL-1β, TNFα, and IL-6 measured in homogenates of mouse cortex using ELISA. Glial markers IBA1, CD45, CD68, and GFAP were assessed by immunohistochemistry. Microglia and amyloid plaques were quantified by immunofluorescence staining followed by confocal imaging. Levels of soluble and insoluble of Aβ40 and Aβ42 were measured by ELISA. The studies of in vivo pharmacodynamic effects on markers of neuroinflammation were complemented by mechanistic studies in the murine microglia BV2 cell line, using live cell imaging techniques to monitor proliferation, migration, and phagocytosis activities.

**Results:**

Intervention with MW150 in KI mice during the established therapeutic time window attenuated the increased levels of IL-1β and TNFα but not IL-6. MW150 treatment also increased the IBA1^+^ microglia within a 15 μm radius of the amyloid plaques, without significantly affecting overall microglia or plaque volume. Levels of IBA1, CD45, CD68, GFAP, and Aβ40 and Aβ42 were not affected by MW150 treatment. MW150 did not significantly alter microglial migration, proliferation, or phagocytosis in BV2 cells.

**Conclusions:**

Our results demonstrate that MW150 at an efficacious dose can selectively modulate neuroinflammatory responses associated with pathology progression without pan-suppression of normal physiological functions of microglia.

**Electronic supplementary material:**

The online version of this article (doi:10.1186/s12974-017-0845-2) contains supplementary material, which is available to authorized users.

## Background

Alzheimer’s disease (AD) is the most common form of dementia, affecting >5.3 million people in the USA alone [1]. Current treatments stabilize symptoms temporarily without slowing progression of the disease [2]. Therefore, there is a pressing need for neuroprotective agents for AD and related dementias.

An array of complementary clinical and preclinical reports implicate discrete aspects of early stage neuroinflammation in disease progression. For example, genome-wide and genetic investigations reveal associations between inflammation-related genes and AD risk (for recent reviews, see [3–5]). Complementary to such associations are experimental outcomes from clinical pathology and animal model investigations that together provide a pathophysiology link between early stage neuroinflammation and AD risk (for reviews, see [6–8]). A specific form of neuroinflammation and dysregulated proinflammatory cytokine production, was shown to be a viable drug discovery pathway if intervention was in early stages when basal cytokine levels were progressively increasing prior to or coincident with synaptic dysfunction [9].

The p38 mitogen-activated protein kinase (MAPK) family of serine-threonine protein kinases, especially p38αMAPK (MAPK14), are key regulators of proinflammatory cytokine production in the brain [10]. Activation of microglia p38αMAPK and the downstream overproduction of proinflammatory cytokines such as TNFα leads to synaptic protein loss, neurite degeneration, and neuronal death in microglia-neuron co-cultures [11]. Activation of p38αMAPK in neurons occurs in response to a variety of CNS disease-relevant stressors, and inhibition of neuronal p38αMAPK can be neuroprotective [12, 13]. Taken in its entirety, the increasing body of evidence is consistent with activation of p38αMAPK activity in both neurons and glia in response to disease-relevant stressors, raising the possibility that selective dosing with a brain-penetrant, isoform-selective p38αMAPK could be a viable approach to disease modification or attenuation of disease progression susceptibility.

The recent description [14] and initial validation of a highly selective p38αMAPK inhibitor, MW150, that is efficacious in two different AD models provided both a mechanistic precedent and a novel candidate for development. Previous efforts at validating p38αMAPK as a viable CNS target used mixed-kinase inhibitors whose results were difficult to interpret or lacked blood brain barrier penetrance and bioavailability to allow exposure to the brain-activated p38αMAPK [15]. The development of MW150 solved those problems. Briefly, MW150 is a unique small molecule drug candidate that is the most highly specific p38αMAPK inhibitor reported to date. MW150 is orally bioavailable, CNS-penetrant, and efficacious in rescuing hippocampal-dependent associative and spatial memory deficits in mouse models of AD-related pathology [14]. MW150 outcomes in a series of pharmacological screens forecast lower risk for development and documented the absence of safety problems that plagued previous CNS p38MAPK inhibitor campaigns. The battery of critical pharmacological and efficacy results qualified this unique, isoform-selective, p38αMAPK inhibitor as a candidate for investigational new drug (IND) development required for first-in-human clinical studies. There is no comparable drug or drug candidate with the combination of documented specificity and safety, in vitro and in vivo pharmacological features, and target recognition [14, 15]. Therefore, it is essential that the glial effects of this exceptional inhibitor with in vivo efficacy be addressed in order to anticipate future use in clinical investigations.

The goal of the current study was to determine in more depth the effects of MW150 on glial biology and proinflammatory cytokine dysregulation in the AD-relevant APP/PS1 knock-in (KI) mouse model previously used to show MW150 efficacy in suppression of hippocampal-dependent associative and spatial memory deficits [14]. To further probe the selective action of MW150, we complemented the in vivo studies by live cell imaging analysis of the murine microglia BV2 cell line to monitor proliferation, migration, and phagocytosis activities. Our results demonstrate that MW150 has a selective role in modulation of neuroinflammatory responses without pan-suppression of normal physiological functions of microglia.

## Methods

### Animals

The AD mouse model we used is the APP^NLh/NLh^ × PS1^P264L/P264L^ KI mouse model originally developed at Cephalon [16]. This double KI mouse line expresses mutant APP and PS1 under the control of the endogenous promoters, and therefore, shows AD pathology without APP or PS1 overproduction. The APP/PS1 KI mice were maintained on a CD-1/129 background; wild-type (WT) control mice were obtained from heterozygous APP/PS1 matings and were maintained as a separate line for >20 generations, as previously described [9].

### Synthesis and use of MW150

MW01-18-150SRM (MW150) was synthesized and characterized as previously reported [14]. For all experiments in this report, MW150 was dissolved in 0.9% sterile NaCl (saline: Hospira NDC 0409-4888-10) and was administered (2.5 mg/kg/day) by intraperitoneal (i.p.) injection to 1112-month-ld APP/PS1 KI mice once daily for 14 days. APP/PS1 KI and WT mice administered saline vehicle i.p. under the same administration paradigm were used as controls.

### Brain tissue harvesting, biochemical, and immunohistochemical and immunofluorescent endpoints

Mice were anesthetized with 5% (v/v) isoflurane prior to transcardial perfusion with ice-cold PBS for 5 min. The brains were then rapidly removed and were fixed or homogenized as previously described [9]. IL-1β, TNFα, IL-6, Aβ40, or Aβ42 levels were measured in cortex homogenates using Meso Scale Discovery (MSD) ELISA, as previously described [9, 17, 18]. Immunohistochemical (IHC) staining was done, and images quantified with the Aperio ScanScope XT digital slide scanner and Aperio ImageScope software positive pixel count algorithm (version 9) as previously described [9, 17]. Primary antibodies used for IHC staining included: rabbit anti-glial fibrillary acidic protein (GFAP) at 1:10,000 dilution (cat# Z0334; Dako); rat anti-CD68 at 1:5000 dilution (cat# MCA1957T; Serotec); and rat anti-CD45 (YW62.3) at 1:10,000 dilution (cat# MA1447081; ThermoFisher Scientific). For the detection of GFAP, a HRP-conjugated goat anti-rabbit IgG was used. For all other primary antibodies, a biotinylated secondary antibody was amplified in avidin-biotin substrate (ABC kit, Vector Laboratories). All sections were developed in 0.5 mg/ml 3,3-diaminobenzidine tetrahydrochloride solution (Sigma, cat# D5637).

Immunofluorescence staining was done following established methods as previously described [19, 20]. Antibodies used included: rabbit anti-IBA1 at 1:200 dilution (cat# 019-19741; Wako); and biotin-labeled mouse anti-Aβ (6E10) at 1:200 dilution (cat# 39340-200, Covance). Primary antibodies were detected by Alexa 488 goat anti-rabbit IgG at 1:200 dilutions (cat# A-11034, Life Technologies) or Alexa Fluor 594 streptavidin (cat# S32356, Life Technologies). Immunofluorescent images were taken on a Nikon C2Plus Confocal Microscope using a 40× objective, at 18 μm range with 0.175 μm step size, 2× zoom, 512 × 512 pixel size, 0.0003 mm/pixel. Imaris software (version 8.1.2: Bitplane AG) was used for 3D reconstructions of the confocal Z-stacks. An observer blind to experimental conditions selected regions of interest in the cortex in a pseudo-randomized fashion using the presence of a 6E10 positive Aβ plaque as the only criterion for selection. The surface creation tool was used to create surfaces for Aβ and microglia. Amyloid plaque surfaces larger than 10,000 voxels were considered “large plaques”. Distance transformation tool (MATLAB; version R2016b MathWorks) was used to create the distance channel from plaques. A surface of 15 μm radius around large plaques was created using the distance channel. We empirically tested a range of different radiuses from 2–300 μm on a test image. The goal was to find a radius that captured the majority of microglia that were plaque associated (i.e., touching), while avoiding microglia that were not in close proximity (i.e., not touching) the plaque. 15 μm was chosen as the radius that best met these criteria.

### BV2 cell culture

The murine microglial BV2 cell line [21] was cultured in DMEM/F12 media (cat#15-090-CV, Mediatech) supplemented with 10% FBS, 100 IU/ml penicillin, 100 μg/ml streptomycin (cat# 30-002-CI, Mediatech), and 2 mM L-Glutamine (cat# 25-005-CI, Mediatech), as previously described [18].

Proliferation, migration, and phagocytosis assays were done as previously described [19]. Cytochalasin D (CytD; cat# C8273, Sigma), an inhibitor of actin polymerization, was used as a positive control. CytD was dissolved in dimethyl sulfoxide (DMSO; cat# D2650, Sigma); therefore, a DMSO control at the same concentration was included in all experiments. As no difference was found between the saline control and the DMSO control, only the saline control values are shown.

For proliferation assays, BV2 cells were plated in a 96-well plate at 5000 cells/well in the presence of saline, DMSO (0.01%v/v), cytD (1 μM), or MW150 (3.75, 7.5, 15 μM). Cell density (image confluence) was recorded every 2–3 h using IncuCyte Zoom Live Cell Imager (Essen Bioscience) with 10× objective and analyzed with IncuCyte Zoom software (Essen Bioscience). Three independent experiments were performed, with eight technical replicates conducted for each experiment.

For phagocytosis assays, BV2 cells in a 96-well plate (5000 cells/well) were incubated with saline, DMSO (0.01%v/v), cytD (1 μM), or MW150 (3.75, 7.5, 15 μM) for 30 min. pHrodo red *E. coli* bioparticles (cat# P35361, ThermoFisher Scientific) were then added to the wells at a final concentration of 400 μg/ml. Fluorescence of the BV2 cells in the red channel was recorded every 30 min using IncuCyte Zoom at 20× objective. Three independent experiments were performed, with four technical replicates conducted for each experiment.

BV2 cell migration was assessed in a scratch wound assay. In a 96-well plate, the WoundMaker (Essen Bioscience) was used to create a strip devoid of cells in the center of each well when the cells were approximately 90% confluent. Saline, DMSO (0.01%v/v), cytD (1 μM), or MW150 (3.75, 7.5, 15 μM) was added to each well, and images were recorded every 2–3 h using IncuCyte Zoom with 10× objective. The average size of the scratch wound that had filled with cells at 12 h post-scratch was determined by the percent confluency in the area left nearly devoid of cells after the scratch wound and normalized to vehicle. Three independent experiments were performed, with eight technical replicates conducted for each experiment.

MW150 inhibition of lipopolysaccharide (LPS)-induced proinflammatory cytokine upregulation in BV2 cells was measured as previously described [14]. Briefly, BV2 cells were plated at a cell density of 2 × 10^4^ in a 48-well plate and incubated for 24 h. Cells were then treated with either saline control or 100 ng/ml of LPS (*Salmonella enterica serotype typhimurium*, cat# L6143, Sigma, 600,000 EU/mg), in the absence or presence of increasing concentrations of MW150. After 16-h incubation, levels of TNFα in the conditioned media were measured by MSD ELISA.

### Statistics

Graphs and statistical analyses were done using GraphPad Prism software version 6.0. A one-way analysis of variance (ANOVA) was used to compare three or more groups. A two-tailed Student’s *t* test was used for comparisons between KI + veh vs. KI + MW150-treated animals and WT + veh vs. KI + veh, as these comparisons were decided a priori to be the only ones of interest. A *p* value < 0.05 was considered significant. Values are expressed as mean ± SEM. Data for all endpoints are available in Additional files 1, 2, 3 and 4: Tables S1-S4.

## Results

### MW150 reduces proinflammatory cytokine levels in the cortex of APP/PS1 KI mice

**Fig. 1:**
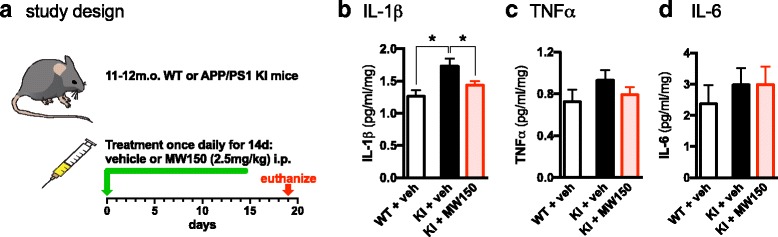
Selective attenuation of proinflammatory cytokines by MW150 administration in APP/PS1 KI mice cortex. **a** 11–12-month-old wild type (*WT*) or APP/PS1 knock-in (*KI*) mice were treated with saline vehicle (*veh*) or 2.5 mg/kg MW150 by intraperitoneal injection (i.p.) once daily for 14 days. **b** IL-1β was increased in KI + veh mice compared to WT + veh mice (*p* = 0.0012), and MW150 treatment of KI mice (KI + MW150) significantly attenuated IL-1β levels compared to KI + veh (*p* = 0.0243) (*F*(2,38) = 6.46; *p* = 0.004). **c** TNFα was elevated in KI + veh mice compared to WT + veh and attenuated in KI + MW150 mice compared to KI + veh; however, these changes were not significant (*F*(2,38) = 1.11; *p* = 0.34). **d** IL-6 was slightly elevated in KI + veh compared to WT + veh mice, and there was no effect of MW150 treatment (*n* = 11 WT + veh; *n* = 14 KI + veh; *n* = 14 KI + MW150). Data are mean ± SEM. Source data is available in Additional file 1: Table S1

Previously, we found that MW150 rescued cognitive function as measured in the radial arm water maze in APP/PS1 KI mice [14]. Here, we investigated endpoints that may be associated with the protective effect of MW150 treatment in this mouse model. In many in vitro and in vivo systems, disease- or injury-induced activation of p38αMAPK and subsequent upregulation of proinflammatory cytokines have been linked to downstream synaptic dysfunction. Therefore, the effect of MW150 treatment on proinflammatory cytokine levels was measured. For the study design, 11–12-month-old WT or KI mice were treated daily for 14 days with saline vehicle (veh) or MW150 (2.5 mg/kg) by i.p. injection. The mice were euthanized at day 19 after the start of treatment (Fig. 1a). As expected, based on our previous studies with the KI mice [9], protein levels of IL-1β in cortex homogenates were markedly elevated in KI + veh mice compared to WT + veh mice (Fig. 1b). MW150-treated KI mice showed significantly reduced IL-1β, with levels approaching those in WT mice (Fig. 1b). A similar pattern of changes was seen with TNFα; however, this change was not statistically significant (Fig. 1c). MW150 did not inhibit IL-6 levels under the conditions used in this experiment (Fig. 1d).

### MW150 treatment does not significantly alter GFAP immunostaining in the cortex of APP/PS1 KI mice

**Fig. 2:**
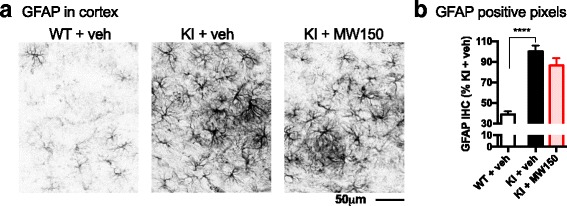
No effect of MW150 on GFAP immunostaining. **a** Representative images of GFAP immunohistochemical (IHC) staining in cortex of WT or APP/PS1 KI mice treated with vehicle (*veh*) or MW150. **b** Digital quantification of GFAP in the cortex was done using the Aperio ScanScope with the entire cortex used as the region of interest. Quantification using the positive pixel algorithm showed a significant increase in GFAP staining in the KI + veh compared to WT + veh (*p* < 0.0001). No significant difference was found between the KI + veh compared to the KI + MW150. (F2,41) = 34.66; *p* < 0.0001). (*n* = 14 WT + veh; *n* = 14 KI + veh; *n* = 14 KI + MW150). Data are mean ± SEM. Source data is available in Additional file 2: Table S2

To investigate whether astrocyte activation might be modulated by MW150 treatment, we measured a marker of reactive astrocytes, GFAP. The APP/PS1 KI mice showed higher levels of GFAP immunostaining than WT mice (Fig. 2a). KI mice treated with MW150 showed a trend of reduced GFAP staining in the cortex compared to KI + veh-treated mice, but this difference did not reach significance (Fig. 2b).

### MW150 treatment does not alter multiple markers of reactive microglia in the cortex of APP/PS1 KI mice

**Fig. 3:**
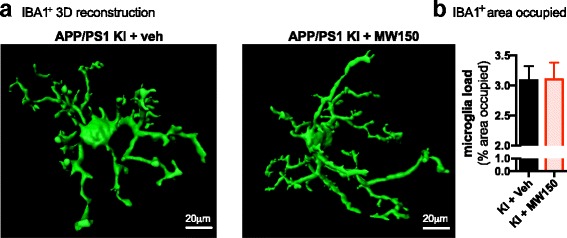
No effect of MW150 on IBA1^+^ microglia volume. **a** Representative 3D surface reconstructions of IBA1^+^ cells generated from confocal microscopic imaging using Imaris software. **b** Mean volume ± standard deviation (SD) of rendered IBA1 cells is shown for the APP/PS1 KI + veh and APP/PS1 KI + MW150 groups. Data represent mean of 3–4 independent z-stacks from each mouse. (*n* = 11 KI + veh; *n* = 14 KI + MW150)

IBA1 is a commonly used pan-marker of microglia and macrophages. Change in morphology (i.e., hypertrophy) of IBA1-positive cells is used as a marker of a reactive microglia response. To determine if MW150 had an effect on the reactive microglia response, we performed immunofluorescent staining for IBA1, followed by confocal microscopy and three-dimensional (3D) reconstruction using Imaris software. For each animal, 3–4 confocal z-stacks were collected. A 3D surface rendering was made of the IBA1 staining, and the total volume of the 3D z-stack that was occupied by the rendered IBA1^+^ staining was determined, for approximately 40–70 microglia per animal. The volume of microglia staining included all IBA1^+^ staining captured in the z-stack including processes not associated with cell bodies. As shown in Fig. 3, there was no difference in the volume of microglia in the KI mice treated with MW150 or vehicle.

**Fig. 4:**
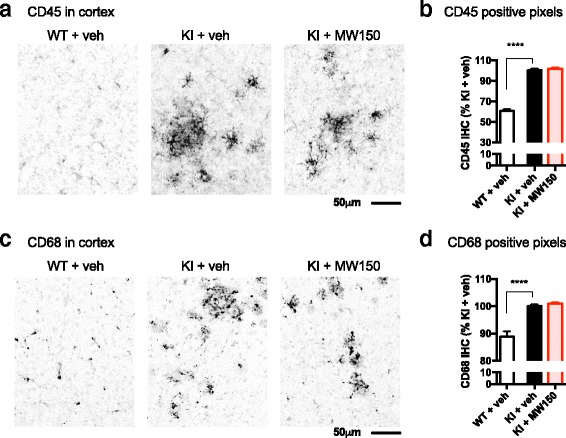
No effect of MW150 on CD45 and CD68 immunostaining in the cortex. **a** Representative images of CD45 IHC in cortex of wild type (*WT*) or APP/PS1 KI mice treated with saline vehicle (*veh*) or MW150. Digital quantification of CD45 in the cortex was done using the Aperio ScanScope, and the positive pixel algorithm. **b** CD45 was significantly increased in the KI + veh compared to the WT + veh treated mice (*p* < 0.0001), with no effect of MW150 treatment. (*F*(2,42) = 211.08; *p* < 0.0001). **c** Representative images of CD68 IHC. **d** CD68 was significantly increased in the KI + veh compared to the WT + veh-treated mice (*p* < 0.0001), with no effect of MW150 treatment. (*F*(2,42) = 28.81; *p* < 0.0001). (*n* = 14 WT + veh; *n* = 14 KI + veh; *n* = 14 KI + MW150). Data are mean ± SEM. Source data is available in Additional file 2: Table S2

To further investigate changes in microglia/macrophage activation, we used two additional markers that are expressed at low levels in the mouse brain without an activating stimulus or pathology (Fig. 4). CD45 is a transmembrane protein that is expressed on all nucleated hematopoietic cells [22]. Its expression on resident microglia is low, but upon stimulation, its expression in microglia is upregulated [23]. IHC staining of CD45^+^ cells in mice cortex was increased in KI mice compared to WT; however, CD45^+^ staining was not significantly altered with MW150 treatment (Fig. 4a. b). CD68 is a glycoprotein that is associated with lysosomes and is therefore linked to activated microglia, macrophages, and other phagocytic cells [24, 25]. CD68 was increased in the KI + veh mice compared to the WT mice, and MW150 treatment of the KI mice did not decrease CD68 staining (Fig. 4c, d).

### MW150 treatment has no significant effect on Aβ plaque volume or on levels of soluble or insoluble Aβ in the APP/PS1 KI mice

**Fig. 5:**
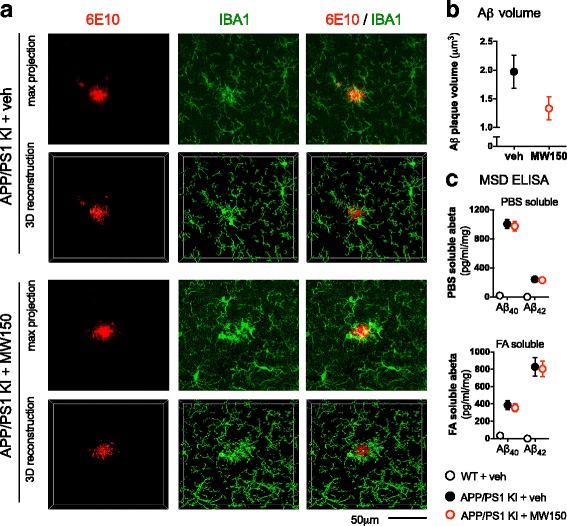
No effect of MW150 on Aβ in APP/KI mice. **a** Aβ and microglia volume were measured using immunofluorescent staining with 6E10 for Aβ and IBA1 for microglia. A z-stack of images were taken using confocal microscopy then were analyzed using the surface tool in Imaris software. Representative confocal images and 3D surface reconstructions with Imaris software are shown. **b** Aβ volume occupied by the surface reconstruction was reduced in KI + MW150; however, the decrease was not significant. The data represents average of 3–4 independent z-stacks from each mouse (*n* = 11 KI + veh; *n* = 14 KI + MW150). **c** PBS- and FA-soluble Aβ40 or Aβ42 levels were measured by MSD ELISA. No significant effect of MW150 treatment was found in the Aβ ELISA. (*n* = 11 WT + veh; *n* = 14 KI + veh; *n* = 14 KI + MW150). Data are mean ± SEM. Source data is available in Additional file 1: Table S1 and Additional file 3: Table S3

We previously reported that MW150 had no effect on Aβ plaque burden in the APP/PS1 KI mice [14], as assessed by IHC staining. To test whether a potential effect of MW150 on Aβ might be revealed with quantitative and more detailed assays, we performed immunofluorescent staining and confocal analysis, as well as quantitative Aβ ELISAs. Aβ plaques were stained with the 6E10 antibody, and microglia were stained with IBA1 (Fig. 5a). 3D reconstructions were generated from the confocal z-stacks using Imaris software, and Aβ plaque volume was calculated. A slight reduction was found in the total volume occupied by Aβ plaques in the KI + MW150 compared to the KI + veh-treated mice, but the difference did not reach significance (Fig. 5b). Measurement of Aβ40 and Aβ42 levels in PBS soluble and formic acid (FA) soluble fractions of APP/PS1 KI mice cortex by quantitative Aβ ELISA showed that MW150 had no effect on Aβ levels (Fig. 5c).

### MW150 treatment increased plaque-associated IBA1^+^ cells but did not significantly increase Aβ inside IBA1^+^ cells in the cortex of KI mice

**Fig. 6:**
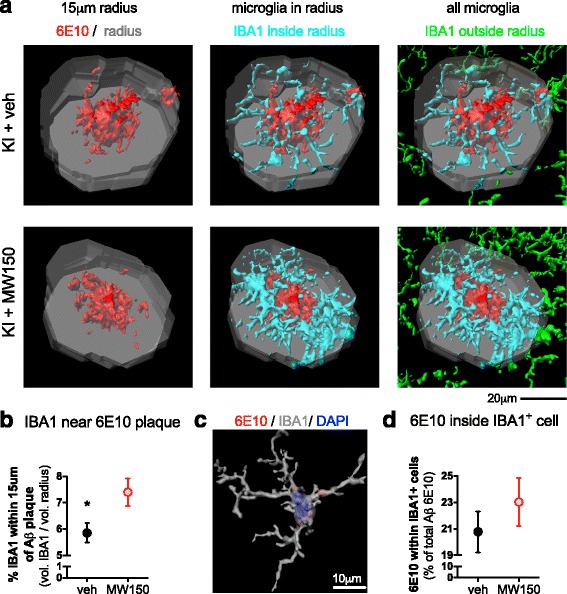
Effect of MW150 treatment on microglia closely associated with Aβ plaques and microglia-internalized Aβ in the cortex of APP/PS1 KI mice. **a** Representative images of Imaris 3D reconstruction of plaques. A region of interest (*ROI*) was generated by expanding the plaque volume by a 15 μm radius from the edge of the large plaques (larger than 10,000 voxels). This 3D ROI (shown in *gray*) included the Aβ plaque, and a region near the plaque. IBA1^+^ cells in this ROI (shown in *cyan*) were surface rendered to create a 3D volume of all IBA1 positive staining in the ROI. The IBA1 positive staining in the 3D ROI distinguishes plaque-associated microglia (shown in *cyan*) compared to microglia away from plaques (shown in *green*). **b** Volume of surface rendered IBA1^+^ cells within 15 μm radius around large plaques was significantly increased in KI + MW150 mice compared to KI + veh treatment (*p* = 0.0397). **c** Representative image of microglia reconstruction with DAPI stained nuclei showing 6E10 staining within IBA1^+^ cell cytoplasm. **d** Microglia-internalized Aβ, as measured by 6E10 staining within surface rendered IBA1^+^ cells, was not significantly different between the KI + MW150 compared to KI + veh. (*n* = 11 KI + veh; *n* = 14 KI + MW150). Data are mean ± SEM. Source data is available in Additional file 3: Table S3

To determine if inhibition of p38αMAPK would affect Aβ-microglial interactions, we tested the effect of MW150 treatment of KI mice on the number of microglia associated with amyloid plaques, and the amount of Aβ internalized by microglia. To quantify the number of plaque-associated microglia, we generated a 3D reconstruction of 6E10^+^ amyloid plaques, with a focus only on 6E10^+^ amyloid plaques larger than 10,000 voxels. Using the distance transformation tool in the Imaris software, we then made a 3D region of interest (ROI) that was 15 μm radius from the edge of the plaque and included the plaque (Fig. 6a). Then, to determine the number of plaque-associated microglia, a surface rendering of the IBA1^+^ cell was generated (as in Fig. 3) for all of the IBA1^+^ staining in this 3D plaque-associated ROI. Quantification of the volume of IBA1^+^ staining showed a significantly increased volume of IBA1^+^ staining surrounding each plaque in KI + MW150 compared to the KI + veh-treated mice (Fig. 6b).

We further investigated whether MW150 had an effect on how much Aβ was internalized by the IBA1^+^ cells. A mask for the 6E10^+^ staining was created, such that only 6E10^+^ staining that was colocalized with the surface rendering of the IBA1^+^ cells was included in the analysis (Fig. 6c). The volume of the 6E10^+^ staining within IBA1^+^ staining was determined as a marker of the amount of Aβ internalized by the IBA1^+^ cells. MW150 treatment resulted in a slight increase in the amount of Aβ internalized by the IBA1^+^ cells compared to KI + veh-treated mice, but the difference was not significant (Fig. 6d).

### MW150 treatment does not impair microglial proliferation, migration, or phagocytosis in BV2 cells

**Fig. 7:**
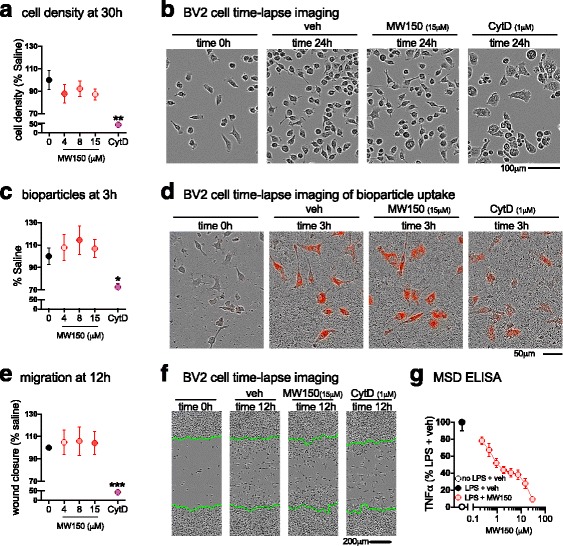
No effect of MW150 treatment on BV2 cell proliferation, migration, and phagocytosis. **a** Average BV2 cell density at 30 h after plating in a 96-well plate at 5000 cells/well. **b** A representative example of the cell density at 24 h after treatment with saline, MW150 (0, 3.75, 7.5, or 15 μM), or cytochalasin D (cytD;1 μM) (mean ± SEM, *n* = 3 independent experiments; 8 technical replicates included for each experiment). **c** Quantification of pHrodo-labeled *E. coli* bioparticles at 3 h after addition of bioparticles. **d** A representative example of the cell bioparticles uptake at 3 h after treatment with saline, MW150 (0, 3.75, 7.5, or 15 μM), or cytD (1 μM) (mean ± SEM, *n* = 3 independent experiments; 4 technical replicates included for each experiment). **e** Average size of scratch wound that is filled with cells, as determined by the percent confluency in the area left nearly devoid of cells after the scratch wound, normalized to veh at 12 h post scratch. **f** Representative images of the scratch wound made (highlighted by *green lines*), at time 0 and 12 h post scratch (mean ± SEM, *n* = 3 independent experiments; 8 technical replicates included for each experiment). **g** MW150 concentration-dependent inhibition of TNFα levels in LPS-stimulated BV2 cells (mean ± SEM, *n* = 1–3 independent experiments; 4 technical replicates included for each experiment). Source data is available in Additional file 4: Table S4

To further investigate the action of MW150 on microglia physiological functions, we used IncuCyte Zoom live cell imaging methods to measure proliferation, migration, and phagocytosis in a mouse microglial BV2 cell line exposed to increasing concentrations of MW150 (3.75 to 15 μM). Proliferation was measured by recording cell density (image confluence) at 30 h after compound treatment. Under the same culture conditions, we previously found [19] that BV2 cells show a linear cell growth rate at this time point. As shown in Fig. 7a, b, there was no significant effect of MW150 on BV2 cell density at any concentration tested. The positive control cytochalasin D (cytD), a known inhibitor of actin polymerization, caused a significant decrease in cell growth and an abnormal cell morphology (Fig. 7a, b).

Next, we assessed if MW150 would affect BV2 cell phagocytic ability, with an assay that uses *E. coli* bioparticles as an indicator of phagocytosis. The bioparticles have a pHrodo dye that is non-fluorescent at neutral pH but becomes fluorescent in the red spectrum when it enters the acidic environment of a phagosome. MW150 (3.75, 7.5, 15 μM) or CytD was added 30 min prior to addition of bioparticles, then fluorescence intensity was captured by IncuCyte Zoom software every 30 min. We have previously reported [19] that in BV2 cells, the fluorescence intensity gradually increases over time and reaches a plateau by about 4 to 5 h after the addition of bioparticles. MW150-treated cells did not show significant differences in fluorescence intensity compared to saline-treated cells (Fig. 7c, d), but there was a trend toward increased phagocytosis with MW150. The positive control, CytD, significantly reduced the uptake of bioparticles by BV2 cells. The noticeable reduction in fluorescence intensity with CytD treatment is illustrated in Fig. 7d.

We used a scratch wound assay to assess the capability of BV2 cells to migrate into an injury area devoid of cell coverage. When BV2 cells reached approximately 90% confluency in a 96-well plate, a scratch wound was made by the Essen Bioscience WoundMaker. Previously we reported [19] that by 24 h the space made by the scratch would be filled with BV2 cells. At 12 h after the scratch wound was made, MW150 treatment did not significantly alter the amount of empty space left by the scratch wound, whereas CytD caused a significant delay in wound closure (Fig. 7e, f).

Because MW150 had no suppressive activity in the proliferation, migration, or phagocytosis assays, it was important to confirm that MW150 was active in the cells. Therefore, as a positive control we tested the ability of MW150 to suppress TNFα production from LPS-stimulated BV2 cells, a pathway known to be regulated by p38αMAPK. We found that MW150 suppressed LPS-stimulated TNFα upregulation with an IC50 of 1.34 to 1.99 μM (95% CI), concentrations well below the range of MW150 (3.75, 7.5, 15 μM) used for the live cell imaging assays.

## Discussion

There are two major findings in this study. First, treatment with an established efficacy dose of MW150 attenuated age-related increases in brain IL-1β and TNFα without affecting the overall amount of microglia or Aβ levels. A surprising finding was that MW150 treatment increased the microglia closely associated with amyloid plaques. Second, MW150 treatment did not suppress protective microglia cell responses such as migration, proliferation, or phagocytosis over concentrations that attenuate proinflammatory cytokine production. These key findings document the selective effect of efficacious doses of MW150 on disease-linked glia processes with retention of responses considered protective.

The results reported here extend our previous validation [14] of MW150 as a candidate for development by showing that MW150 in the efficacious dose range suppresses IL-1β and TNFα overproduction in vivo in the APP/PS1 KI mice*.* We did not detect a change in IL-6 levels, consistent with the prevailing view that IL-6 cytokine induction generally occurs via signaling pathways independent of p38αMAPK [26].

We previously reported [14] that MW150 showed no detectable effects on amyloid plaque burden in either the APP/PS1 KI mice or the APP/PS1 transgenic mice assessed by standard immunohistochemistry with 6E10 anti-Aβ antibody. We further extended the analyses here by using quantitative Aβ ELISA and confocal microscopy. Aβ ELISA demonstrated that MW150 had no detectable effect on the levels of PBS-soluble or formic acid-soluble Aβ40 and Aβ42 in the APP/PS1 KI mouse cortex, consistent with our previous study that reported no change in plaque load by immunohistochemistry [14]. Confocal microscopy analysis showed that MW150 treatment of APP/PS1 KI mice had no significant effect on the total Aβ plaque volume in the cortex and on the amount of internalized Aβ inside each microglia. However, even though the results did not reach significance, there was a trend toward decreased Aβ plaque volume and increased Aβ inside microglia in the APP/PS1 KI mice treated with MW150. These results are also consistent with a small increase in bioparticle phagocytosis activity seen in BV2 cells after MW150 treatment. Overall, our findings raise the intriguing possibility that MW150 stimulates microglia phagocytosis. However, these data must be interpreted with caution because the in vivo results with Aβ plaque volume did not reach statistical significance, and in vitro results in the BV2 cell line with bioparticle phagocytosis cannot be directly equated to Aβ phagocytosis in vivo. It will be important in future studies to test potential effects of MW150 on microglia phagocytosis in more detail. Nevertheless, it is clear that MW150 did not impair the ability of the plaque-associated microglia to phagocytose Aβ and did not lead to a significant change in Aβ40 or Aβ42 levels.

An unexpected finding in the results reported here was that MW150 treatment significantly increased the microglia in close proximity to amyloid plaques. Whether this reflects an effect of MW150 on microglia migration is unclear. There was no statistically significant effect of MW150 on migration of BV2 cells in response to a scratch wound, but similar studies with migration to amyloid deposits were not done because of limited responsiveness of BV2 cells to synthetic Aβ. The functional significance of the increase in plaque-associated microglia is also not known, but a logical hypothesis to test in future studies is whether there is a relationship between modulation of p38αMAPK signaling, and the ability of microglia processes to surround amyloid plaques and promote their compaction. A demonstrated linkage would provide mechanisms whereby microglia could decrease compaction (p38αMAPK mediated) or increase compaction (e.g., TREM2 mediated; [27]). The unexpected finding, therefore, raises the potential of subtle effects of p38αMAPK inhibition on microglia—plaque interactions that will require further exploration.

There is some general confusion in the literature about the pharmacodynamic effects of p38αMAPK inhibitor drug candidates. Specific to this report, there is an unresolved issue in terms of the relationship between p38αMAPK and amyloid pathology in preclinical mouse models of AD. Our results here and elsewhere [14] using multiple experimental approaches clearly show that the isoform-specific p38αMAPK inhibitor MW150 does not significantly affect overall Aβ levels or plaque burden. However, previous studies with some p38αMAPK inhibitors that hit multiple kinases, such as VX-745 and CNI-1493, report inhibition of Aβ production or amyloid plaque deposition [28–30]. Whether the effects on these inhibitors on amyloid reflect engagement of targets other than p38αMAPK [31, 32], such as Abl [33], is not known. Further, a full evaluation of the differences is limited by the type of animal model used in efficacy and pharmacodynamic endpoint analyses. For example, APP/PS1 transgenic mouse models that overexpress APP exhibit high levels of amyloid, whereas the APP/PS1 KI mouse uses endogenous promoters and demonstrates progressive AD pathology without the forced APP overproduction. Regardless, future clinical studies are required to resolve any therapeutic significance of these preclinical pharmacodynamic differences.

## Conclusions

In summary, our results demonstrate the selective effects on glial inflammatory responses to treatment with MW150, a unique, isoform-selective, p38αMAPK inhibitor drug candidate that attenuates cognitive impairment in AD-relevant mouse models. The findings show that MW150 has a selective role in modulation of neuroinflammatory responses without pan-suppression of normal physiological functions of microglia. The refined insight into glial biology presented here, combined with the safety and efficacy pharmacological profile of MW150 [14], reinforce the need to move this novel therapeutic candidate into clinical development for AD and related disorders.

## Additional files

Additional file 1: Table S1. Cytokines and Aβ MSD. Source data for cytokine data in Fig. 1 and Aβ data in Fig. 5. (XLS 67 kb)

Additional file 2: Table S2. IHC assays. Source data for GFAP data in Fig. 2 and CD68 and CD45 data in Fig. 4. (XLS 63 kb)

Additional file 3: Table S3. Confocal Imaris assays. Source data for microglia load in Fig. 2, plaque load in Fig. 5, and microglia/plaque data in Fig. 6. (XLS 59 kb)

Additional file 4: Table S4. BV2 assays. Source data for Fig. 7. (XLS 80 kb)

## Abbreviations

AD: Alzheimer’s disease
ANOVA: Analysis of variance
CytD: Cytochalasin D
DMSO: Dimethyl sulfoxide
FA: Formic acid
GFAP: Glial fibrillary acidic protein
i.p.: Intraperitoneal
IHC: Immunohistochemical
KI: Knock-in
LPS: Lipopolysaccharide
MAPK: Mitogen-activated protein kinase
MSD: Meso Scale Discovery
MW150: MW01-18-150SRM
ROI: Region of interest
veh: Vehicle
WT: Wild-type
